# Development of a Chemically Modified Sensor Based on a Pentapeptide and Its Application for Sensitive Detection of Verbascoside in Extra Virgin Olive Oil

**DOI:** 10.3390/ijms232415704

**Published:** 2022-12-11

**Authors:** Irina Georgiana Munteanu, Vasile Robert Grădinaru, Constantin Apetrei

**Affiliations:** 1 Department of Chemistry, Physics and Environment, Faculty of Sciences and Environment, “Dunărea de Jos” University of Galaţi, 47 Domneasca Street, 800008 Galaţi, Romania; 2 Faculty of Chemistry, Alexandru Ioan Cuza University, 11 Carol I Bd., 700506 Iasi, Romania

**Keywords:** verbascoside, screen-printed electrode, pentapeptide, graphene oxide, cyclic voltammetry, olive oil

## Abstract

In addition to their antioxidant and antimicrobial action in functional foods, beverages, and in some dermato-cosmetic products, olive phenolic compounds are also recognized for their role in the prevention of diabetes and inflammation, treatment of heart disease and, consequently, of the numerous chronic diseases mediated by the free radicals. In recent years, attention has increased, in particular, regarding one of the most important compound in extra virgin olive oil (EVOO) having glycosidic structure, namely verbocoside, due to the existence in the literature of numerous studies demonstrating its remarkable contribution to the prophylaxis and treatment of various disorders of the human body. The purpose of this study was the qualitative and quantitative determination of verbascoside in commercial EVOOs from different regions by means of a newly developed sensor based on a screen-printed carbon electrode (SPCE) modified with graphene oxide (GPHOX), on the surface of which a pentapeptide was immobilized by means of glutaraldehyde as cross-linking agent. The modified electrode surface was investigated using both Fourier-transform infrared spectroscopy (FTIR) and scanning electron microscopy (SEM) methods. This newly developed sensor has shown a high sensibility compared to the unmodified electrode, a low detection limit (LOD) of up to 9.38 × 10^−8^ M, and a wide linearity range between 0.1 µM and 10.55 µM. The applicability of the modified sensor was confirmed by detecting verbascoside in ten different EVOOs samples using the cyclic voltammetry (CV) method, with very good results. The validation of the electroanalytical method was performed by using the standard addition method with very good recoveries in the range of 97.48–103.77%.

## 1. Introduction

In recent years, the study of GPHOX-based materials has been expanded especially in terms of electrochemical applications. Underlying the importance of GPHOX-based materials in electrochemistry are their unique characteristics, such as very good chemical stability, high hydrophilicity, high dispersity in various solvents and ease of functionalization [1]. Typically, GPHOX is obtained by a simple wet oxidative exfoliation process of graphite using strong oxidants [2] followed by the manufacture of flexible GPHOX thin films by simple liquid phase processes such as the casting technique [3,4], vacuum filtration [5] and spray coating [6]. The physical and chemical properties of GPHOX offer promising applications for chemical sensors [7], mechanical [8] and electronic [9], energy storage [10], optoelectronic devices [11], nanoelectronics [12] and biotechnology [13]. A series of characteristics of GPHOX, including the high dispersibility in water, but also in organic solvents, the very good surface-to-volume ratio, the existence of a wide range of surface-bound reactive functional groups (mainly epoxy and hydroxyl groups) [14], gives fascinating properties to GPHOX-based materials, making them attractive for electrochemical studies and applications [15]. In addition, GPHOX has been shown to facilitate direct electron transfer from enzymes and proteins to the surface of electrodes modified with this type of nanostructured material [16]. These characteristics, in addition to providing the possibility of explaining the intrinsic thermodynamic and kinetic electron transfer properties of the electroactive species at the interfaces of GPHOX-based electrodes, also open a new path to new electrochemical sensors and new electroanalytical methods [17].

Peptides have been used as sensitive components for the fabrication of novel sensors in view of a number of advantages they present, including relatively simple synthetic protocols [18], diverse structures [19] and the possibility of use as highly selective substrates for enzymes [20]. Peptides are smaller versions of proteins [21]. They are short strings (i.e., between two and fifty) of amino acids that are linked by peptide bonds [22]. Peptides are short strings of amino acids, whereas amino acids are likewise the structure squares of proteins. It implies that peptides are little sisters of proteins [23]. Since these compounds do not often generate a signal that can be measured directly in response to a binding process, conjugation with a signal marker is an efficient method to transform the binding information into a measurable signal [24]. Different materials, such as gold nanoparticles, have been used as signal markers [25], quantum dots [26], graphene [23,27], lanthanide chelates [28], and also electrochemical markers [29]. The use of peptides is a promising approach in view of the fact that these biomolecules can be used as biorecognition elements when connected with electrochemical sensors [30], representing an encouraging alternative for the study of both antioxidant activity and antioxidant compounds in different pharmaceutical or food products.

**Figure 1:**
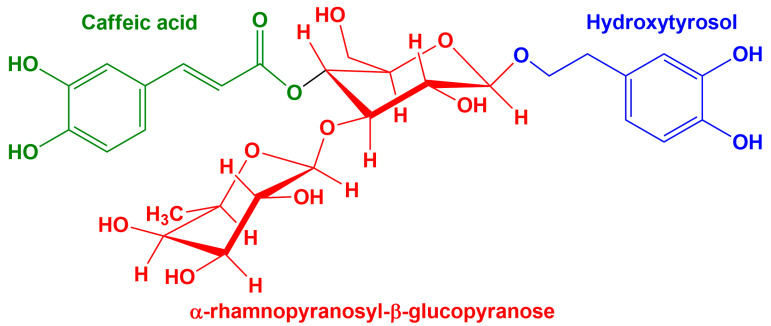
The chemical structure of verbascoside glycoside.

The Mediterranean diet is a primarily plant-based eating plan that includes a daily intake of whole grains, fruits, vegetables, beans, and other legumes, nuts, herbs, and spices, the consumption of which ensures protection against heart disease and cancer [31]. Olive oil is recommended as the primary added fat, replacing other oils and fats (butter, margarine). Olives and olive oil have been shown to contain phenolic compounds with strong biological activities, including antioxidant activity [32]. The composition of olive oil is primarily triacylglycerols (~99%) and secondarily free fatty acids, mono- and diacylglycerols, and an array of lipids such as hydrocarbons, sterols, aliphatic alcohols, tocopherols, and pigments. An abundance of phenolic and volatile compounds is also present. Some of these compounds contribute to the unique character of the oil. Hydroxytyrosol and tyrosol are the most abundant phenolic alcohols in olives [33]. Flavonoids include flavone glycosides such as luteolin-7-glucoside and rutin, as well as anthocyanins. Verbascoside (Figure 1), a derivative of hydroxycinnamic acid, is also found in these samples and is one of the most potent antioxidants in olive oils; its unique antioxidant activity is due to the synergistic effect of the combination of the two diphenolic constituents, namely caffeic acid and hydroxytyrosol [34]. More concentrated in the olive fruit, but also present in the leaves, this compound has an important antioxidant activity, therefore, has a direct impact on skin health, preventing oxidative damage associated with wrinkling, thinning skin or dehydration [35].

In the specialized literature, there are numerous studies on the development and evaluation of methods for the analysis, isolation and identification of phenolic compounds from olive oils [36,37]. Among the analytical methods for the quantification of polyphenols in olive oil is the Folin–Ciocalteau spectrophotometric assay for the evaluation of phenolic compounds content [38,39] and also high-performance liquid chromatography (HPLC) [40,41] for the identification and quantification of individual phenolic compounds.

Recently, due to their high sensitivity, low cost, and the possibility of miniaturization and automation, a number of sensors and biosensors have been described in the literature, their simplicity favoring their use for olive oil analysis over classical methods [42,43]. Among electrochemical methods, voltammetric techniques have been successfully used to detect phenolic compounds in aqueous solutions and in different food matrices, for example, tea, wine, beer, and, not least, olive oil [44].

**Figure 2:**
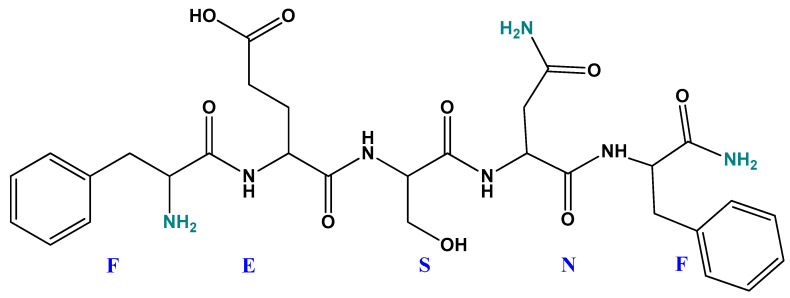
Chemical structure of pentapeptide under study.

In this study, a direct electrochemical method capable of assessing and quantifying the verbascoside content in different EVOO samples is described with minimal sample preparation using an SPCE modified with a GPHOX composite film on the surface of which a pentapeptide (NH_2_-FESNF-CO-NH_2,_ sequence where F-phenylalanine, E-glutamic acid, S-serine, and N-asparagine), the sequence of which is found in the primary structure of the lysozyme in egg white, has been immobilized (Figure 2). The proposed method is simple, fast and offers a promising alternative to more complex analytical techniques. To the best of our knowledge, until now, there is no study related to the detection of verbascoside with a GPHOX-peptide modified sensor, and in the future, research should be conducted on the use of this device for the detection of other types of samples, such as pharmaceuticals in different dosage forms, dietary supplements or biological samples such as human serum.

## 2. Results and Discussions

### 2.1. Electrode Characterisation

The construction of a robust sensor by modifying its surface with pentapeptide solution and fixing it by cross-linking involves obtaining analytically important parameters, including sensitivity, lifetime, detection, and quantification limits [45].

To observe the changes using pentapeptide in the commercial SPCE/GPHOX, its active surface was analyzed by means of two techniques, the FTIR spectrometric method and SEM, respectively.

#### 2.1.1. FTIR Spectrometric Method

**Figure 3:**
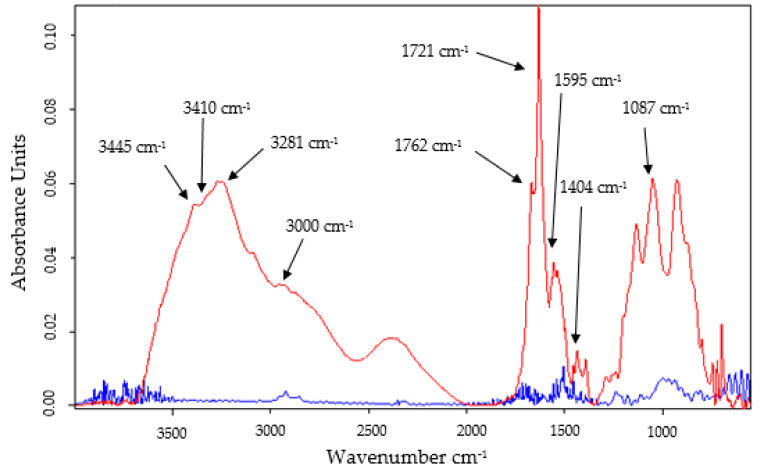
FTIR spectra for SPCE/GPHOX (blue line) and SPCE/GPHOX-Pentapeptide/(red line).

FTIR spectra (Figure 3) were recorded for both the single electrode, SPCE/GPHOX, and the modified electrode, SPCE/GPHOX-Pentapeptide, to confirm peptide film formation on the sensor surface and also to define the characteristic peaks of GPHOX-related functional groups containing oxygen, such as epoxy, carbonyl and hydroxyl.

The different oxygen-containing functional groups in the GPHOX structure have been identified by the characteristic vibrational modes of the epoxy (C-O-C) group (1230–1320 cm^−1^) [43], a stretch band corresponding to the C=O group in the carboxyl group at 1721 cm^−1^ [46,47], a deformation vibration band related to O-H at 1404 cm^−1^ [48], respectively a stretching vibration 1087 cm^−1^ belonging to the alkoxy group C-O [49]. Moreover, in the FTIR spectrum of GPHOX, due to extensive oxidation, GPHOX has a strong and broad O-H stretching vibration band at 3410 cm^−1^ [50], characteristic of GPHOX due to the existence of many hydroxyl groups in its structure [51].

The FTIR method is one of the few techniques that can also be applied to the structural characterization of peptides in different media. Thus, the type I amide bands found in the range 1600–1700 cm^−1^ were the most intense absorption bands, indicating that a β-sheet conformation is present in the structure of the peptide film formed at the sensor surface [52]. Along with these, the band at 1595 cm^−1^ can be attributed to additional interactions occurring in the fibrils, such as protofilament packing [53,54]. The existence of peaks corresponding to the amide supports the presence of the pentapeptide on the whole electrode surface. These peaks are not present in the case of the unmodified electrode, i.e., SPCE/GPHOX [55]. The band observed at 1762 cm^−1^ can be attributed to the C=O stretching mode of the carboxyl group of either asparagine or glutamate residue or C=O from the amide group located on the C-terminal amino acid [56]. Moreover, the stretching vibration corresponding to free or non-hydrogen bonding -NH_2_ groups occurs at 3445 cm^−1^ [57] and at 3281 cm^−1^ [58,59], respectively, and the absorption corresponding to wave number 3000 cm^−1^ corresponds to the O-H stretching vibration of the hydroxyl group in the serine structure [60].

#### 2.1.2. Morphological Characterisation Using SEM

**Figure 4:**
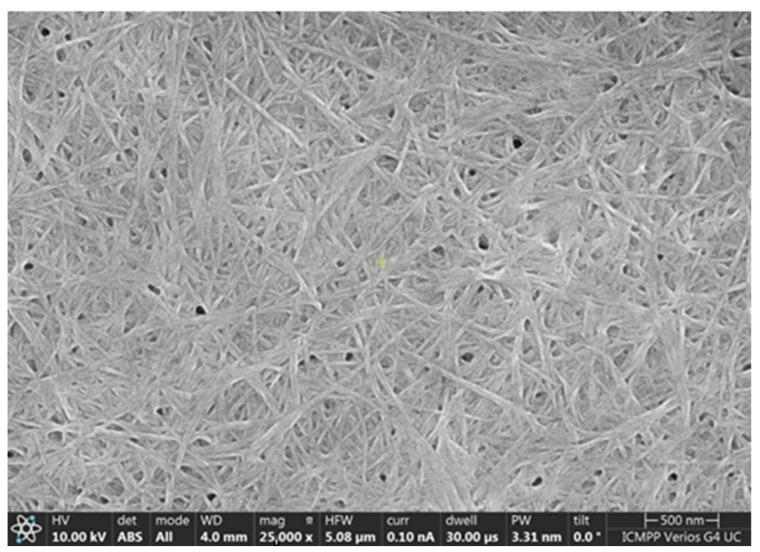
SEM image representing the active surface of SPCE/GPHOX-Pentapeptide.

The SEM image (Figure 4) shows the morphology of the active sensor surface. Three-dimensional arrangements consisting of continuous fibers with good homogeneity are observed, showing a homogeneity of the composite nanofilm surface. On the other hand, in Figure 4, the presence of GPHOX sheets is not clearly evident. They are covered by fibers, and this is evidence of GPHOX incorporation in the peptide fibrillary network [59].

### 2.2. Preliminary Studies for the Characterisation of SPCE/GPHOX-Pentapeptide

Taking into account our previous work [23], where it was demonstrated that the optimal pH value for electrochemical determinations at which a stable signal was obtained was 6.5, the determinations in this study were performed in solutions obtained by dissolving in 0.1 M PBS at pH 6.5. Moreover, in the same study, the optimal amount of peptide required for sensor modification was determined using different volumes of the peptide solution, demonstrating that the anodic peak was more intense and better defined when using a volume of 20 µL [23]. Therefore, the same volume of 20 µL of the pentapeptide solution was also used in the present study to obtain the modified sensor.

**Figure 5:**
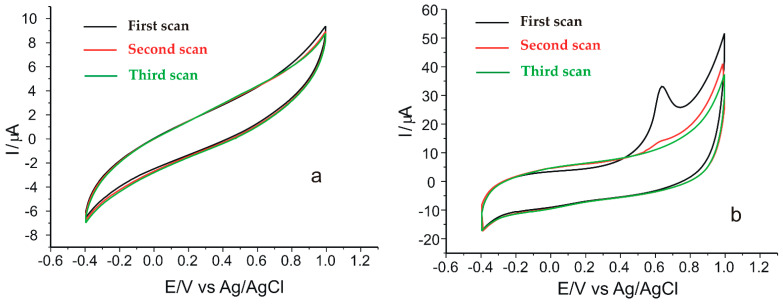
CVs registered in 0.1 M PBS solution by SPCE/GPHOX (**a**) and SPCE/GPHOX-Pentapeptide (**b**). Scan rate 0.05 V·s^−1^. Three successive cycles.

Further preliminary analyses consisted of evaluating the electrochemical behavior of SPCE/GPHOX and SPCE/GPHOX-Pentapeptide in 0.1 M PBS at pH = 6.5. According to previous studies [23,60,61], the potential range in which a stable signal was obtained was from −0.4 to 1.0 V, proving to be optimal for the new peptide-based sensor described in the present study. The signal was stable after three cycles without interference or background noise, suggesting that there was no contamination of the active surface during the modification steps. Hence, the same potential range was used in the study of the kinetics of the electrochemical reaction of verbascoside. At the same time, the lack of contamination of the active surface of the electrode can also be demonstrated by the fact that when immersing SPCE/GPHOX in 10^−1^ M PBS solution, the CVs do not show oxidation or reduction peaks in the studied potential range (Figure 5a).

Upon immersion of the SPCE/GPHOX-Pentapeptide sensor in 0.1 M PBS solution (Figure 5b), the CVs recorded show the electrode signal changing during the first cycle, with the signal stabilizing after three cycles. In the case of the modified electrode, the oxidation peak appears more intense at the first scan at a potential value of 0.64 V (Ipa = 32.74 µA). At the second and third scan cycles, the oxidation peaks are evident but are not as intense as that present at the first scan. Moreover, in all three successive scans, the reduction peaks can be seen at the same potential and having approximately the same intensity (Epc = −0.02 V, Ipc = –9.58 µA).

### 2.3. Electrochemical Properties of SPCE/GPHOX-Pentapeptide in K_4_[Fe(CN)_6_]/K_3_[Fe(CN)_6_] Solution

K_4_[Fe(CN)_6_] exhibits redox activity, and this can be evidenced by CV, when an anodic and a cathodic peak is obtained due to the reversible oxidation of the ferrocyanide ion to ferricyanide, occurring at the electrode surface [62].

**Figure 6:**
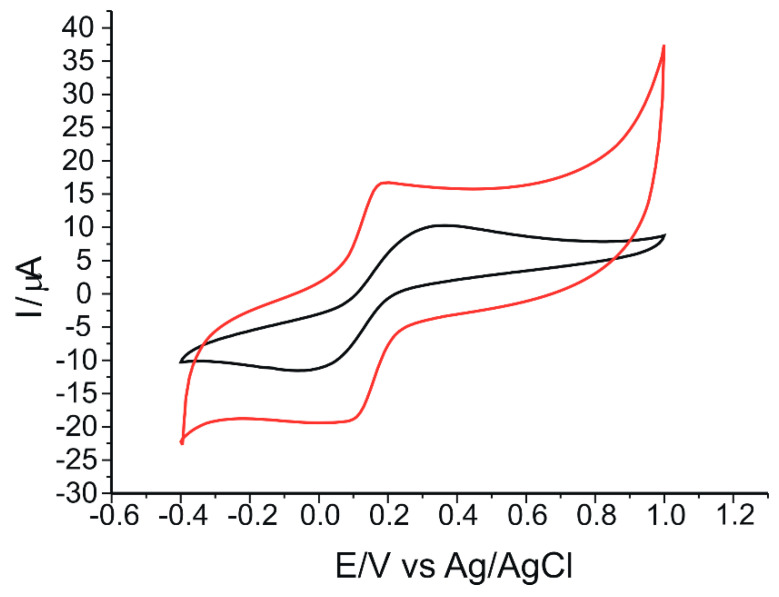
CVs of SPCE/GPHOX (black line) and SPCE/GPHOX-Pentapeptide (red line) immersed in 10^−3^ M K_4_[Fe(CN)_6_]/K_3_[Fe(CN)_6_]–0.1 M PBS solution, at the scan rate of 0.05 V·s^−1^.

The voltammetric responses of the sensors immersed in a solution containing 10^−3^ M K_4_[Fe(CN)_6_]/K_3_[Fe(CN)_6_] dissolved in 0.1 M PBS, pH = 6.5 recorded in the same potential range were well defined and reproducible at a scan rate of 0.05 V·s^−1^ (Figure 6). It can be seen that a pair of redox peaks, one anodic and one cathodic due to the ferrocyanide/ferricyanide redox process, is present in both sensors but of different intensities and potentials.

Voltammetric parameters achieved from the CVs of the two sensors immersed in 10^−3^ M K_4_[Fe(CN)_6_]/K_3_[Fe(CN)_6_]–0.1 M PBS solution are shown in Table 1.

As can be seen, the peptide-modified electrode showed the highest degree of reversibility, with the separation between anodic and cathodic peaks being smaller than for the unmodified electrode and the Ipc/Ipa ratio greater than 1 (1.13). This demonstrates that the reaction process for the modified electrode is quasi-reversible. Both electrodes show similar electrochemical behavior, with almost double sensitivity in the case of the pentapeptide-modified sensor, according to the obtained parameters, and can be successfully used in further determinations.

**Figure 7:**
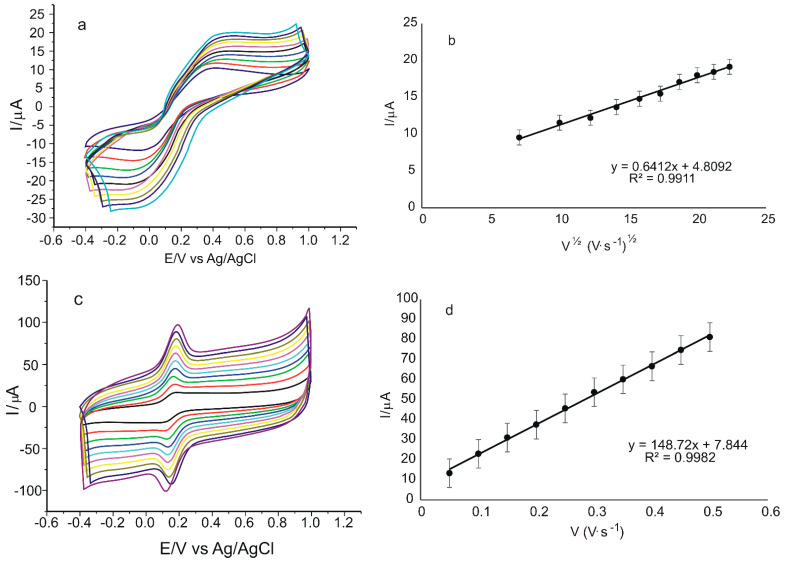
CVs of SPCE/GPHOX (**a**) and SPCE/GPHOX-Pentapeptide (**c**) immersed in 10^−3^ M K_4_[Fe(CN)_6_]/K_3_[Fe(CN)_6_]–0.1 M PBS solution at pH = 6.5 recorded at scan rates between 0.05 and 0.5 V·s^−1^. Linear dependence of Ipa and square root of scan rate in the case of SPCE/GPHOX (**b**) and linear dependence of Ipa and scan rate in the case of SPCE/GPHOX-Pentapeptide (**d**).

Another important parameter in the electrochemical determination of redox processes using CV is the scan rate. Thus, the influence of scan rate on the voltammetric responses of the sensors was obtained by recording the CVs of the two electrodes in 10^−3^ M K_4_[Fe(CN)_6_]/K_3_[Fe(CN)_6_]–0.1 M PBS solution. These results are shown in Figure 7.

In Figure 7a,c, it can be seen that as the applied scan rate increases, the anodic and cathodic peak currents also increase linearly, indicating a quasi-reversible oxidation reaction [63]. The measured values for the anodic peak currents were used to determine the linear dependence equation of Ipa on the square root of the scan rate, v^1/2^, which indicated a typical diffusion-controlled reaction [64] obtained for the unmodified sensor (Figure 7b). The dependence between Ipa and scan rate, v, using the same experimental conditions, was a linear dependence in the case of the modified peptide-based sensor (Figure 7d), which is typical for the process controlled by the adsorption of the electroactive species [65]. Therefore, the modification of the pentapeptide sensor had a favorable effect on the reaction kinetics at the electrode surface, also creating an optimal environment for an analyte-sensitive layer interaction and, therefore, a fast electron transfer, resulting in a change of the rate-determining factor of the oxido-reduction process in the case of SPCE/GPHOX-Pentapeptide [66].

### 2.4. Electrochemical Sensor Responses in Verbascoside Solution

The next step of the present study was to analyze the behavior of both the modified and the unmodified sensor in verbascoside solution using CV.

**Figure 8:**
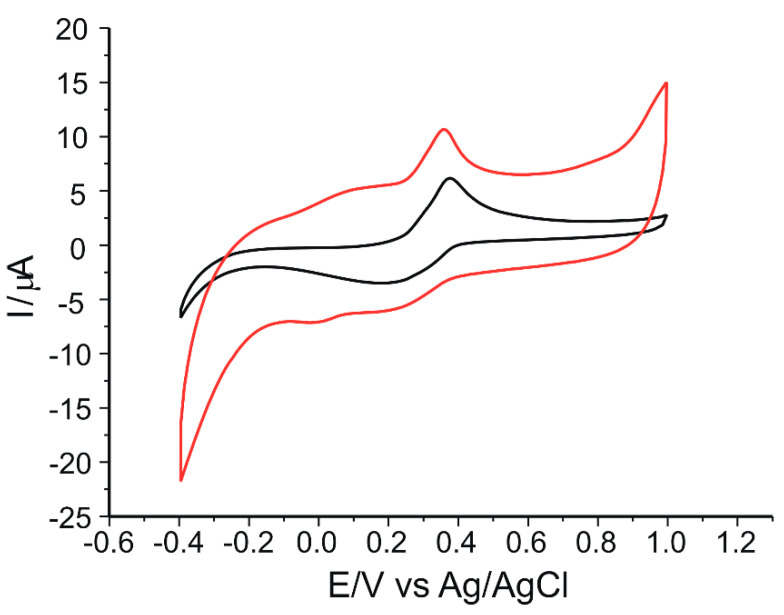
CVs of SPCE/GPHOX (black line) and SPCE/GPHOX-Pentapeptide (red line) immersed in 10^−4^ M verbascoside–0.1 M PBS solution (pH 6.5). Scan rate: 0.05 V·s^−1^.

Figure 8 shows, by comparison, the response of the two sensors when immersed in a solution of verbascoside 10^−4^ M-PBS 0.1 M (pH = 6.5). In order to obtain a stable sensor response, three successive cycles in the optimized potential range (−0.4 V to 1.0 V) were required. The CVs shown in Figure 8 are obtained after the stabilization of the signals.

In the case of the unmodified sensor, the cyclic voltammogram shows an anodic oxidation peak at the potential value of Epa = 0.38 V and a cathodic reduction peak at the potential value of Epc = 0.21 V.

Regarding the behavior of the peptide-modified sensor upon immersion in verbascoside solution, it is different from the unmodified sensor, which is evident in the corresponding voltammogram (red line) in Figure 8. Thus, the oxidation of verbascoside occurs in two steps, showing two anodic and two cathodic peaks of different intensities and potentials. The first oxidation peak occurs at the potential value of Epa1 = 0.08 V, followed by the second, well-defined oxidation peak at the potential value of Epa2 = 0.35 V. When scanning in the negative direction, the first reduction peak appears at a potential value of Epc2 = 0.22 V, and the second at a potential value of Epc1 = 0.01 V. The differences between the anodic and cathodic peak potentials, Epa1 – Epc1 = 0.07 V and Epa2 – Epc2 = 0.13 V, are in agreement with the corresponding value for a reversible reaction involving two electrons at each stage [67].

**Figure 9:**
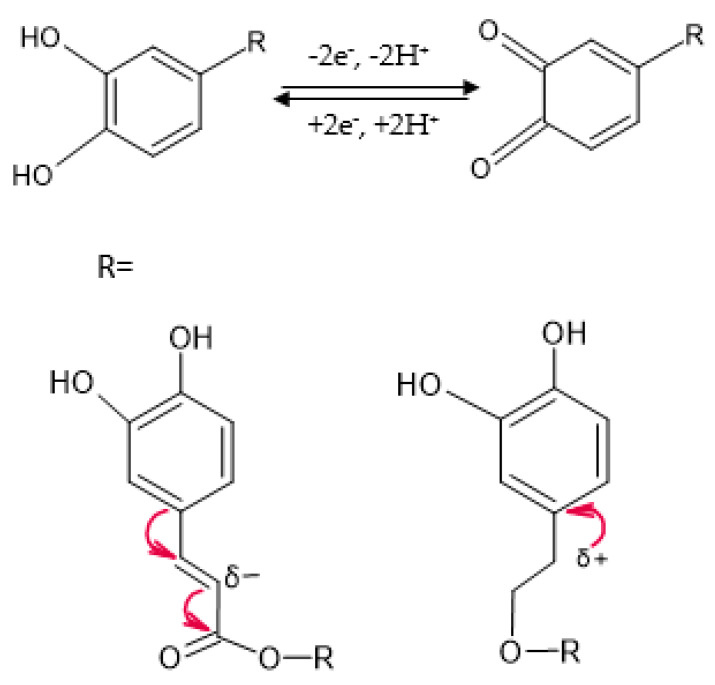
Oxidation mechanism of catechol moiety in verbascoside structure.

The electrochemical oxidation of verbascoside follows a mechanism of compounds with catecholic structure, i.e., a two-electron and two-proton transfer process, since, in the chemical structure of this compound, there are two catecholic moieties corresponding to caffeic acid and hydroxytyrosol [68] (Figure 9). The two catechol fragments have different electron densities due to the functional groups to which they are bound. Since the carboxyl group is not directly linked to the aromatic ring, the electron-attracting inductive effect will be supported by the double bond. Moreover, the positive inductive effect of the alkyl chain increases the electron density on the aromatic ring, lowering the electrochemical oxidation potential. Therefore, we can deduce that the first pair of redox peaks is due to the caffeic acid residue, and the second pair of peaks appear due to the oxidation of hydroxytyrosol.

The electrochemical parameters achieved from the CVs of the two sensors immersed in 10^−4^ M verbascoside solution are presented in Table 2.

The reduced value of the first anodic peak potential in the case of SPCE/GPHOX-Pentapeptide indicates a lower activation energy of the oxidation process, being influenced by the adsorption of verbascoside on pentapeptide predominantly through hydrogen bonds [67] and through π–π stacking interactions between pentapeptide and verbascoside aromatic rings [69]. Therefore, in the case of this sensor, the electron transfer occurring at the active surface is faster compared to the unmodified sensor [70].

From the determinations made so far, it can be deduced that the higher anode peak intensity in the modified sensor indicates a higher sensitivity of the sensor [71] and the lower potentials at which peaks related to the oxidation-reduction process of verbascoside occur in the case of the same modified sensor may suggest a better selectivity of the sensor for this analyte [72].

**Figure 10:**
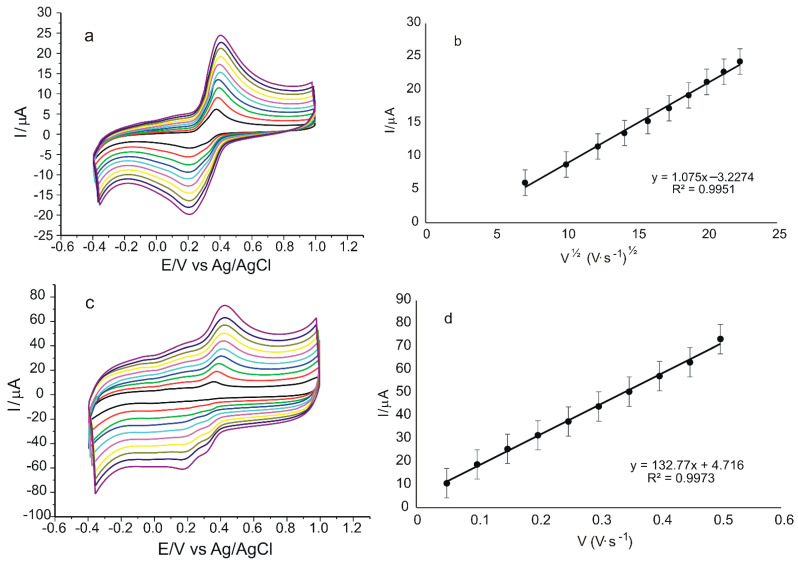
CVs of SPCE/GPHOX (**a**) and SPCE/GPHOX-Pentapeptide (**c**) immersed in 10^−4^ M verbascoside–0.1 M PBS at pH = 6.5 recorded at scan rates between 0.05 and 0.5 V·s^−1^. Linear dependence between Ipa and square root of scan rate in the case of SPCE/GPHOX (**b**) and linear dependence between Ipa and scan rate in the case of SPCE/GPHOX-Pentapeptide (**d**).

The next step was the study of the influence of scan rate on the voltammetric responses of the two sensors in a solution of 10^−4^ M verbascoside-0.1 M PBS, pH = 6.5, applying scan rates in the range 0.05–0.5 V·s^−1^ (Figure 10). Since the anodic peaks are higher and better defined compared to the catodic ones, the dependence of Ipa on the scan rate or square root of the scan rate will be studied.

By increasing the scan rate, it was obvious that the maximum oxidation potentials for both sensors (Figure 10a,c) were shifted toward more positive values and the reduction potentials toward more negative values. However, in the case of SPCE/GPHOX-Pentapeptide the Epa1-Epc1 and Epa2-Epc2 differences were always close to the theoretical value of 0.13 V corresponding to a reversible two-electron, two-proton mechanism, even for high scan rates. In the case of SPCE/GPHOX, as the scan rate increases, the intensity of the oxidation peak current increases linearly with the square root of the scan rate, consistent with oxidation limited by the diffusion of an active species in solution [73]. On the other hand, in the case of SPCE/GPHOX-Pentapeptide, as the scan rate increases, the intensity of the oxidation peak current also increases linearly with the scan rate, consistent with the electron-transfer-controlled oxidation process [74]. Moreover, the anodic peak intensities were higher in the modified sensor compared to those obtained with the unmodified sensor, the presence of peptide on the sensor surface significantly improving its sensitivity. The explanation can be related to the ability of some compounds to interact simultaneously or competitively with mixtures of proteins or peptides, the nature and mechanisms of the interaction depending on various environmental factors such as ionic strength, pH or concentration of the compounds on the one hand, and on the other hand on the structural characteristics, i.e., the polarity of the molecules, steric conformation and size of the molecules [75]. Compounds with a higher molecular weight but also with a higher number of hydroxyl groups, such as verbascoside, have a higher number of sites for interaction with peptides [76]. These interactions can occur via non-covalent bonds, i.e., hydrogen bonds or van der Waals forces [77] with the formation of complex structures, the assembly process often requiring a combination of multiple interaction sites [78]. Furthermore, the presence of interactions between the supramolecular structure, i.e., the peptide and the compound of interest, can influence the kinetics of the oxidation-reduction process, increasing the rate of electron transfer to the surface of the modified sensor [79] and also the result of a synergistic potentiation interaction [80].

Taking into account all the above, it is found that the same electrochemical behavior is maintained for both sensors as in the previous determinations in electroactive 10^−3^ M K_4_[Fe(CN)_6_]/K_3_[Fe(CN)_6_]-PBS 0.1 M solution, demonstrating, once again, the role and influence of the modification of the sensor with the pentapeptide on the kinetics of the reaction at the electrode, respectively, on the process of the electron transfer.

### 2.5. Development of Calibration Curve

The concentration of the solutions to be analyzed is of major importance in the response of a voltammetric sensor. The dependence between anodic peak currents and the concentration of the compound of interest, i.e., verbascoside, allows current intensity measurement to be used for quantitative applications.

**Figure 11:**
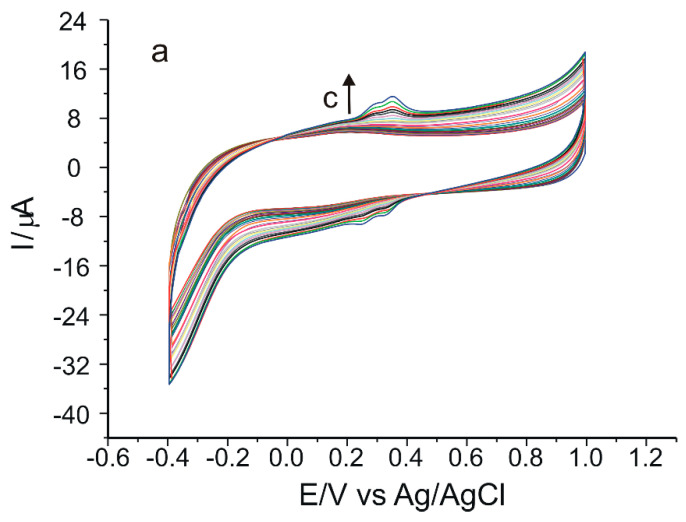

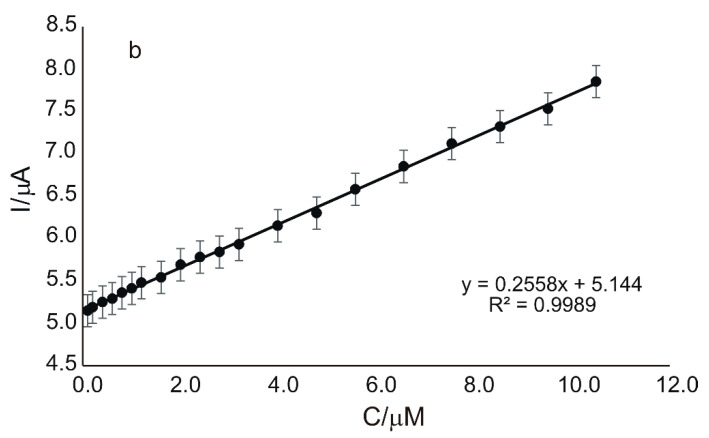
CVs recorded for SPCE/GPHOX-Pentapeptide with the concentration between 0.1 µM and 10.55 µM verbascoside (**a**); Linear dependence between Ipa and verbascoside concentration in the range 0.1–10.55 µM (**b**).

Therefore, to determine the influence of verbascoside concentration on the SPCE/GPHOX-Pentapeptide response, CVs were recorded in verbascoside solutions of different concentrations obtained by dissolving in 0.1 M PBS solution. The electrochemical sensor responses recorded by CV are shown in Figure 11a, and in Figure 11b it can be seen the linear dependence between the Ipa and verbascoside concentration in the linearity range 0.1–10.55 µM when using SPCE/GPHOX-Pentapeptide.

As shown in Figure 11a, a linear relationship was obtained between peak current and verbascoside concentration in the range of 0.1–10.55 µM. Exceeding this concentration, the disappearance of linearity was observed, following a plateau phase in which, although the concentration is still increasing, the intensity of the anodic peak current remains constant in value. This means that all active centers on the sensor surface are involved in the electrochemical reaction [81].

The equation of the calibration curve can help us calculate the limit of detection (LOD) and the limit of quantification (LOQ) of the pentapeptide-modified sensor [82]. These were calculated using the equations LOD = 3σ/m; LOQ = 10σ/m, where σ is the standard deviation (SD) of the current recorded in the control sample (in this case, the PBS solution 0.1 M), and m is the slope of the calibration curve [83].

The values achieved for SPCE/GPHOX-Pentapeptide are: LOD = 9.38 × 10^−8^ M and LOQ = 3.12 × 10^−7^ M. Considering that these values obtained are similar or lower than those reported in the literature [84,85], this sensor was used for the quantitative determination of verbascoside in real samples, i.e., samples of different EVOO.

### 2.6. Accuracy of the Method

The accuracy of the method was evaluated by the standard addition method on samples of known concentration [86]. The percentage recovery of verbascoside added to the sample was evaluated by the relationship between the experimentally determined concentration and the corresponding theoretical concentration [87]. The closer the recovery percentage is to 100%, the more accurate the technique will be for subsequent determination of verbascoside in commercial EVOO samples. [88]. The results obtained, presented in Table 3, show this closeness, confirming that the method is suitable for the objective proposed.

### 2.7. Stability, Reproducibility, Repeatability and Interference Studies

The stability of the SPCE/GPHOX-Pentapeptide was evaluated by performing 30 measurements at regular intervals (one day) for one month using a 10^−4^ M verbascoside solution, using the CV method. Between determinations, the sensor was stored in the refrigerator at 4 °C in a hermetically sealed box. The results showed no significant differences between the anodic currents recorded on different days, with coefficients of variation less than 5%, confirming that the sensor is stable and can be used in electroanalysis.

The reproducibility of the developed method was investigated by preparing five different sensors, using a 10^−4^ M verbascoside solution for performing the test. The calculated RSD value for the Ipa depicted for all five sensors was 3.5%, demonstrating good reproducibility of sensor development.

Tests for repeatability study were performed in a 50 µM verbascoside-0.1 M PBS solution. The value of the coefficient of variation for the anodic peak determined in five consecutive measurements in the same solution did not exceed 2.5%. Between measurements, the SPCE/GPHOX-Pentapeptide was rinsed with 0.1 M PBS solution, pH = 6.5. Therefore, the sensor can be used repeatedly for the determination of verbascoside.

Under optimal experimental conditions, the effect of possible interferents in the form of organic compounds predominantly found in EVOO, such as tyrosol, hydroxytyrosol and oleuropein, on the quantification of verbascoside was evaluated using CV as a detection method. The limit of tolerance was defined as the maximum concentration of interfering compounds that results in a relative error of ±5% for the quantitative determination of verbascoside. The achieved values, presented in Table 4, show that the peaks related to the presence of our compound of interest do not change significantly upon the addition of interferents.

Tolerance limits were calculated with the following results: for oleuropein 5 × 10^−5^ M, and for tyrosol and hydroxytyrosol it was 2 × 10^−5^ M.

Taking into account all the above, it can be deduced that the method described is capable of detecting and quantifying the substance of interest, namely verbascoside, in the presence of chemically related substances, thus being considered specific.

### 2.8. Quantitative Determination of Verbascoside in EVOO

To validate the SPCE/GPHOX-Pentapeptide sensor in the analysis of verbascoside from real samples, ten samples of different EVOOs were selected and subjected to analysis using the CV method for determination. Measurements were performed in the potential range between −0.4 V and 1.0 V, applying a scan rate of 0.05 V·s^−1^.

**Figure 12:**
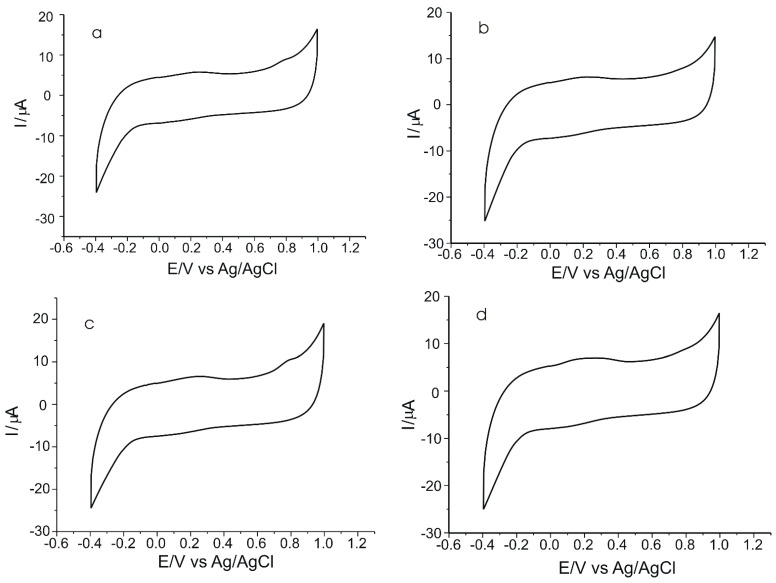
CVs of SPCE/GPHOX-Pentapeptide immersed in solutions obtained from the four EVOOs selected for the study: (**a**) Regina, (**b**) Mazza, (**c**) Minerva, (**d**) Costa D’Oro L’extra.

Figure 12 presents, as an example, the CVs of the pentapeptide-modified sensor immersed in solutions containing extracts from four of the ten EVOOs selected for determination.

In all four voltammograms shown in Figure 12, there is an anodic peak related to the presence of verbascoside in the matrices, on the basis of which this compound was quantified in the respective EVOO samples. Thus, for the calculation of the amount of verbascoside in each sample, the slope of the calibration line obtained by the voltammetric method was used, and the obtained values are shown in Table 5. All analyses were performed in triplicate.

The values obtained for the 10 samples of EVOO on the concentration of verbascoside are close, the highest value being obtained for Costa D’Oro L’extra (Italy).

The precision of the method expressed as relative standard deviation (RSD) was near ±2%, indicating the accuracy of this method.

### 2.9. Determination of the Antioxidant Activity of Verbascoside by DPPH Method; Correlation between Sensor Response and Spectrophotometric Measurements to Determine Antioxidant Activity

This assay provides information on the antioxidants’ capacity to donate electrons or hydrogen atoms scavenging the DPPH free radical [89]. The scavenging capacity of antioxidants toward DPPH free radicals is quantified by the decrease in absorbance at 517 nm [90].

In the first step of the determination, 0.1 mM DPPH stock solution was freshly prepared by dissolving the DPPH reagent in 96% ethanol and kept at room temperature in the dark. Subsequently, 0.5 mL volumes were measured from each sample solution (the same solutions used in the electrochemical measurements) and added to 3 mL of DPPH solution. These were kept at room temperature for 20 min, and the absorbances were then measured at 517 nm against ethanol.

For all 10 samples, the percentage reduction capacity of DPPH radicals was calculated according to the following equation [91]:

$$\%\;Inhibition=\left(\frac{AD-AE}{AD}\right)\times 100$$

where AD is the absorbance of the control solutions (in the absence of antioxidant agent), and AE is the absorbance of the test solutions.

The results obtained are presented as the average of the percentage inhibition values for all sample solutions tested and are included in Table 6.

Table 6 shows that the highest percentage inhibition value was obtained for Costa D’Oro L’extra (Italy), this result being in agreement with the value obtained in the quantitative determination of verbascoside in EVOO samples, where the highest amount of verbascoside contained per kilogram EVOO was also achieved for this matrix.

The next step of the study was to establish correlations between the electrochemical signals provided by the modified sensor and the data obtained from the spectrophotometric determination of antioxidant activity by the DPPH method.

Partial Least Squares regression (PLS) was used as a prediction technique to correlate the voltammetric data obtained by the pentapeptide-modified sensor with the antioxidant activity quantified as the capacity of free radical scavenging. The independent variables (X matrix) were the entire CVs obtained with the modified sensor, and the dependent variable was the percentage of DPPH inhibition. The normalization of the data was performed by the Standard Deviation method.

The results of PLS1 regression models were presented in the form of the dependences between predicted antioxidant activity results towards measured antioxidant activity results.

**Figure 13:**
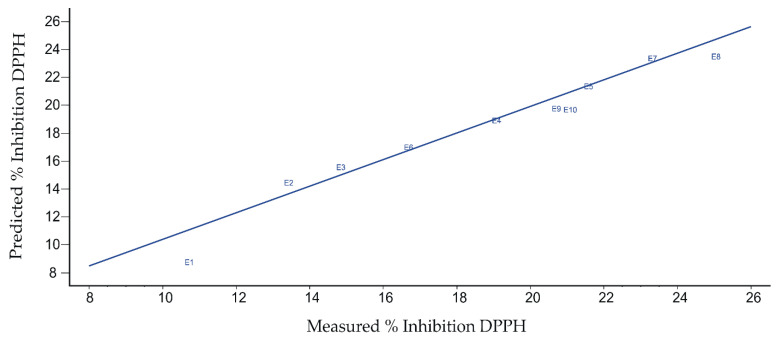
Plot of predicted inhibition from the voltammetric data vs. measured inhibition obtained from DPPH spectrophotometric method.

Figure 13 shows the dependence plot between the percentage inhibition value of DPPH free radical predicted from voltammetric data and the values measured by the spectrophotometric method.

Table 7 shows the quantitative data obtained from the CV-DPPH regression model. It is noted that both calibration and validation values indicate good model performance (correlation coefficient close to 1). In addition, low values of the root mean square error at calibration (RMSEC) and root mean square error at prediction (RMSEP) were obtained for the spectrophotometric method.

It can be appreciated that from the data obtained with the pentapeptide-modified sensor by means of CV, it is possible to estimate accurately the antioxidant activity of the oils expressed as the inhibition capacity of the free radical DPPH.

## 3. Materials and Methods

### 3.1. Chemicals and Solutions

Monosodium phosphate (NaH_2_PO_4_) and disodium phosphate (Na_2_HPO_4_), reagents purchased from Sigma-Aldrich (St. Louis, MO, USA), were used to prepare 0.1 M phosphate buffer solution (PBS), the supporting electrolyte in the electrochemical measurements performed All solutions were prepared with ultra-pure water, MilliQ water (resistivity 18.2 MΩ·cm) obtained from a water purification system (Milli-Q Simplicity^®^, Bedford, MA, USA). pH adjustment to 6.5 was performed by adding 0.1 M H_3_PO_4_ or 0.1 M NaOH. PH measurement is performed using a pH meter from WTW instruments, Weilheim, Germany.

Verbascoside powder of analytical purity was purchased from Sigma-Aldrich, St Louis, MO, USA. A stock solution of verbascoside of concentration 10^−4^ M, prepared by completely dissolving the powder in 0.1 M PBS, was used for electrochemical analysis.

Pentapeptide, of >95% purity (sequence NH_2_ -FESNF-CO-NH_2_, where F is phenylalanine, E is glutamic acid, S is serine, and N is asparagine) was purchased from ProteoGenix, Schiltigheim, France. Peptide purity and retention time were confirmed by RP-HPLC chromatography on a Dionex UltiMate 3000 UHPLC system (Thermo Scientific, Waltham, MA, USA).

Analytically pure compounds structurally similar to verbascoside (oleuropein, tyrosol and hydroxytyrosol) used for the interference studies were also purchased from Sigma-Aldrich (St. Louis, MO, USA).

To obtain the modified sensor (SPCE/GPHOX-Pentapeptide), the SPCE modified with a GPHOX film (SPCE/GPHOX) (purchased from Metrohm DropSens, Oviedo, Spain) was used, onto the surface of which the pentapeptide solution was added and cross-linked with glutaraldehyde. The pentapeptide solution used had a concentration of 10 mg/mL.

The 0.1 mM DPPH (2,2-diphenyl-1-picrylhydrazyl) stock solution was prepared by weighing 0.0036 g DPPH reagent (purchased from Sigma-Aldrich) and dissolving in 100 mL 96% ethanol (Sigma-Aldrich). The resulting solution was kept at room temperature and in the dark until use.

### 3.2. Electrodes and Equipment

A Biologic SP 150 potentiostat/galvanostat (Bio-Logic Science Instruments SAS France) coupled with EC-Lab Express software operating in Windows was used to record, characterize and optimize the electrode signals. Three electrodes were connected to this device: the Ag/AgCl/KCl_3M_ reference electrode, the platinum (Pt) wire auxiliary electrode, and the SPCE working electrode, simultaneously introduced into the electrochemical cell.

The Partner AS 220/C/2 analytical balance was used for weighing the substances, and the most efficient way to dissolve the substances and homogenize the suspensions was by means of an Elmasonic ultrasonic bath (Carl Roth GmbH, Karlsruhe, Germany). The pH meter used for pH measurement was Inolab pH 7310, WTW instruments, Weilheim, Germany.

FTIR spectra were acquired with a Bruker ALPHA FT-IR spectrometer (BrukerOptik GmbH, Ettlingen, Germany) using OPUS software (BrukerOptik GmbH, Ettlingen, Germany) in the wavenumber range between 4000 and 500 cm^−1^, using the attenuated total reflectance (ATR) method as sample exposure mode. The ZnSe crystal was carefully cleaned with ultrapure water and isopropanol between measurements. The background was the spectrum obtained in the air.

The surface morphology of the samples was examined with a Verios G4 UC scanning electron microscope (Thermo Scientific, Waltham, MA, USA) equipped with an energy-dispersive X-ray spectroscopy analyzer (Octane Elect Super SDD detector (AMETEK, Tokyo, Japan). The samples were coated with 10 nm platinum using a Leica EM ACE 200 coating system (Leica Microsystems, Vienna, Austria) to provide electrical conductivity. Then, the samples were fixed with double adhesive tape on the cylindrical conductor supports. SEM investigations were performed in High Vacuum mode using a secondary electron detector (Everhart–Thornley detector, ETD) at an accelerating voltage of 5 kV.

For the spectrophotometric method based on the reaction of antioxidant compounds with the free radical DPPH, sample absorbances were measured using a Rayleigh UV2601 UV/Vis Double Beam Spectrophotometer (Beijing Beifen-Ruili Analytical Instrument, Beijing, China).

### 3.3. Obtaining the Chemically Modified Sensor

**Figure 14:**
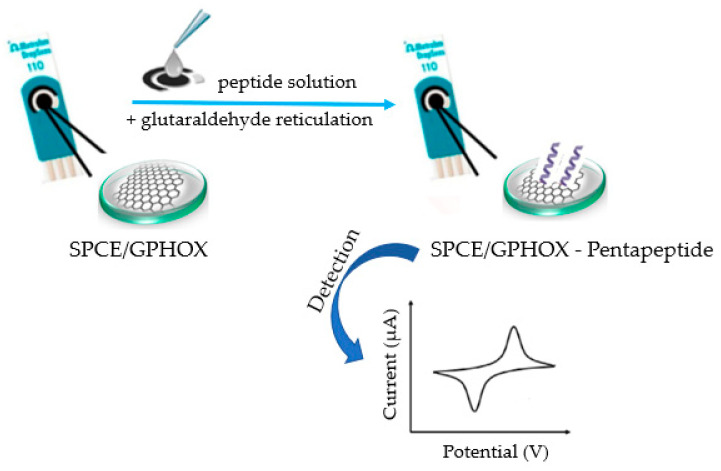
Preparation process of SPCE/GPHOX-Pentapeptide sensor.

For the preparation of the SPCE/GPHOX-Pentapeptide sensor, several steps were carried out. Initially, a 20 µL peptide solution (5 µL in each step) was added to the SPCE/GPHOX surface by the drop-and-dry method sequentially, in four steps, with drying pauses. Drying was carried out at room temperature for 30 min. The addition of the pentapeptide solution was performed using an Eppendorf micropipette. The next step was to hold the sensor over a container of 5 mL 2% glutaraldehyde for 1 min, the glutaraldehyde vapor ensuring the immobilization of the peptide on the electrode surface, a process known as cross-linking. The resulting sensor was stored at 4 °C until use, for a maximum of 72 h [92]. Figure 14 shows the steps in the preparation of the chemically modified sensor.

### 3.4. Methods of Analysis

In the present study, in order to highlight the oxidation-reduction processes taking place on the electrode surface and also to validate the results obtained, the CV method was performed as the electroanalytical technique. This is one of the most widely used methods to characterize electrochemical systems, providing both qualitative and quantitative information about the system studied. CV can provide information on the mechanism of a chemical reaction and the kinetics of the process, and it is also possible to determine the antioxidant properties of active compounds, which depend on their chemical structure and redox properties [93].

### 3.5. Real Samples and Preparation of the Solutions to Be Analysed

To study the sensitive properties of the sensor, 10 extracts from different EVOOs obtained by liquid–liquid extraction were analyzed [94]. An amount of 5 g of each oil was mixed with 10 mL methanol–water solution (40:10, *v/v*). The hydromethanolic extracts were separated using a separation funnel, after which 5 mL methanolic extract was added to 45 mL 0.1 M PBS of pH 6.5, which was the sample analyzed with the sensor made in this study.

Table 8 shows all the commercial samples of olive oils studied.

### 3.6. Antioxidant Activity (DPPH Free Radical Scavenging Activity)

The study of antioxidant activity using this spectrophotometric assay is based on the decolorization of the stable DPPH radical, strongly colored purple-red, by an antioxidant substance. The absorbance measurement is made at 517 nm, the decolorization being an indicator of antioxidant efficacy [95]. Applying this method provides an easy and rapid way to determine antioxidants by spectrophotometry, and various chemical compounds or natural products with antioxidant activity can be evaluated.

### 3.7. Data Analysis

PLS is a method that reduces the variables used to predict to a smaller set of predictors, then is used to perform a regression [96]. PLS1 corresponds to the case where there is only one dependent variable. The PLS1 regression model was used to establish correlations between electrochemical sensor responses and antioxidant activity determined by the DPPH spectrophotometric method (% inhibition degree was the dependent variable).

Multivariate data analysis was performed using Matlab, Excel, and Unscrambler.

## 4. Conclusions

Verbascoside is a pharmacologically active compound with much recent evidence supporting its biological and safety activities [97], having a special structure with many reactive sites [98], and the determination and quantification of this compound in EVOO samples is of real use, considering the health benefits that the consumption of olive oil as a food product can provide.

Therefore, this study reported, for the first time, the fabrication of an electrochemical sensor by modifying a GPHOX-based SPCE with a pentapeptide containing the NH_2_-FESNF-CO-NH_2_ sequence, used for the accurate determination of verbascoside from real matrices with complex composition, i.e., samples of different EVOOs. Applying CV as a detection technique has demonstrated very good analytical performance with applicability in laboratory practice. Kinetic studies of electrochemical processes confirmed the superiority of the modified SPCE/GPHOX-Pentapeptide sensor over the unmodified SPCE/GPHOX sensor, obtaining with SPCE/GPHOX-Pentapeptide a wide range of linearity (0.1–10.55 µM) and a low detection limit (9.38 × 10^−8^ M), respectively. In addition, the influence of other related compounds on the voltammetric response was reduced due to the favorable specificity of the newly developed sensor. Moreover, the antioxidant activity of the compound of interest was achieved by DPPH spectrophotometric assay, thus asserting the antioxidant activity of verbascoside in EVOO samples. Additionally, a good correlation between the results of the DPPH assay and CV was obtained.

The method developed and described in this work has a number of advantages, including good precision, sensitivity, time and low cost of analysis. The development of this peptide-based sensor may also prove effective in quality control of other types of products, namely pharmaceuticals, cosmetics and dietary supplements, representing a challenge for future research in the two related fields of healthcare and pharmaceuticals.

## Figures and Tables

**Table 1:** The values of the parameters obtained from the CVs of the two sensors immersed in 10^−3^ M K_4_[Fe(CN)_6_]/K_3_[Fe(CN)_6_] solution (the electrolyte support was 0.1 M PBS of pH 6.5).

Sensor	Ipa ^1^ (µA)	Ipc ^2^ (µA)	Ipc/Ipa	Epa ^3^ (V)	Epc ^4^ (V)	E_1/2_ ^5^ (V)	ΔEp ^6^ (V)
SPCE/GPHOX	10.19	−10.82	1.06	0.33	0.01	0.170	0.32
SPCE/GPHOX-Pentapeptide	16.77	−19.07	1.13	0.18	0.09	0.135	0.09

^1^ Current of the anodic peak; ^2^ Current of the cathodic peak; ^3^ Potential of the anodic peak; ^4^ Potential of the cathodic peak; ^5^ half-wave potential; ^6^ ΔEp = Epa – Epc.

**Table 2:** The values of the parameters obtained from the CVs of the two sensors immersed in 10^−4^ M verbascoside-0.1 M PBS solution of pH 6.5.

Sensor	Epa1 (V)	Epa2 (V)	Ipa1 (µA)	Ipa2 (µA)	Epc1 (V)	Epc2 (V)	Ipc1 (µA)	Ipc2 (µA)
SPCE/GPHOX	0.38	-	6.05	-	0.21	-	−3.46	-
SPCE/GPHOX-Pentapeptide	0.08	0.35	4.79	10.74	0.01	0.22	−7.20	−5.22

**Table 3:** Analysis of verbascoside recovery using the standard addition method.

Theoretical Concentration (µM)	Discovered Concentration (µM)	Recovery %
1.59	1.55	97.5
2.39	2.48	103.8
3.99	3.93	98.7
5.57	5.61	100.8
6.56	6.66	101.7

**Table 4:** Interference of some organic compounds on verbascoside detection.

Interfering Compound	Concentration of the Interfering Compound (M)	Recovery %	RSD % (±%)
Tyrosol	10^−5^ M	101.09	1.22
Hydroxytyrosol	10^−5^ M	103.18	1.31
Oleuropein	10^−5^ M	102.85	1.28

**Table 5:** Concentrations of verbascoside (*n* = 3) in commercial EVOO obtained by the voltammetric method.

EVOO Samples	mg/kg Verbascoside Achieved by CV	RSD (±%)
Pietro Coricelli	1.36	0.02
TopSeller Oil	1.42	0.03
Regina	1.38	0.01
Mazza	1.41	0.03
Olitalia	1.49	0.04
Costa d’Oro Il Grezzo	1.54	0.04
Minerva	1.55	0.06
Costa D’Oro L’extra	1.72	0.05
Monastiri	1.36	0.02
Rivano Olio	1.39	0.03

**Table 6:** Determination of antioxidant activity of the studied EVOO samples.

EVOO Samples	% Inhibition-DPPH
Pietro Coricelli	10.3
TopSeller Oil	13.1
Regina	14.5
Mazza	18.7
Olitalia	21.2
Costa d’Oro Il Grezzo	16.3
Minerva	22.9
Costa D’Oro L’extra	24.7
Monastiri	20.3
Rivano Olio	20.6

**Table 7:** PLS results of CV-DPPH regression models in calibration and validation.

**Calibration**	**CV-DPPH**
Slope	0.954
Offset	0.847
Correlation	0.977
RMSEC	0.939
**Validation**	
Slope	0.930
Offset	1.271
Correlation	0.956
RMSEP	1.285

**Table 8:** Name and country of provenance of EVOO samples.

No.	Oils Denomination	Country of Provenience
1	Pietro Coricelli	Italy
2	TopSeller Oil	Spain
3	Regina	Italy
4	Mazza	Italy
5	Olitalia	Italy
6	Costa d’Oro Il Grezzo	Italy
7	Minerva	Greece
8	Costa D’Oro L’extra	Italy
9	Monastiri	Greece
10	Rivano Olio	Italy

## Data Availability

Not applicable.

## References

[1] Akbi H., Mekki A., Rafai S., Touidjine S., Boudina N., Bekkar Djeloul Sayeh Z. (2022). Phenomenological Description of the Thermal Reduction Kinetics in Graphene Oxide Films. Mater. Chem. Phys..

[2] Dreyer D.R., Park S., Bielawski C.W., Ruoff R.S. (2010). The Chemistry of Graphene Oxide. Chem. Soc. Rev..

[3] Akbi H., Yu L., Wang B., Liu Q., Wang J., Liu J., Song D., Sun Y., Liu L. (2015). Effect of Reducing System on Capacitive Behavior of Reduced Graphene Oxide Film: Application for Supercapacitor. J. Solid State Chem..

[4] El-Kady M.F., Strong V., Dubin S., Kaner R.B. (2012). Laser Scribing of High-Performance and Flexible Graphene-Based Electrochemical Capacitors. Science.

[5] Yeh C.-N., Raidongia K., Shao J., Yang Q.-H., Huang J. (2015). On the Origin of the Stability of Graphene Oxide Membranes in Water. Nat. Chem..

[6] Pham V.H., Cuong T.V., Hur S.H., Shin E.W., Kim J.S., Chung J.S., Kim E.J. (2010). Fast and Simple Fabrication of a Large Transparent Chemically-Converted Graphene Film by Spray-Coating. Carbon.

[7] Muniyalakshmi M., Sethuraman K., Silambarasan D. (2020). Synthesis and Characterization of Graphene Oxide Nanosheets. Mater. Today Proc..

[8] Zhu S.-E., Krishna Ghatkesar M., Zhang C., Janssen G.C.A.M. (2013). Graphene Based Piezoresistive Pressure Sensor. Appl. Phys. Lett..

[9] Saxena S., Tyson T.A. (2010). Interacting Quasi-Two-Dimensional Sheets of Interlinked Carbon Nanotubes: A High-Pressure Phase of Carbon. ACS Nano.

[10] Choi B.G., Yang M., Hong W.H., Choi J.W., Huh Y.S. (2012). 3D Macroporous Graphene Frameworks for Supercapacitors with High Energy and Power Densities. ACS Nano.

[11] Bao Q., Loh K.P. (2012). Graphene Photonics, Plasmonics, and Broadband Optoelectronic Devices. ACS Nano.

[12] Li F., Jiang X., Zhao J., Zhang S. (2015). Graphene Oxide: A Promising Nanomaterial for Energy and Environmental Applications. Nano Energy.

[13] Yan J.-A., Chou M.Y. (2010). Oxidation Functional Groups on Graphene: Structural and Electronic Properties. Phys. Rev. B.

[14] Chen D., Feng H., Li J. (2012). Graphene Oxide: Preparation, Functionalization, and Electrochemical Applications. Chem. Rev..

[15] Perrozzi F., Prezioso S., Ottaviano L. (2014). Graphene Oxide: From Fundamentals to Applications. J. Phys. Condens. Matter.

[16] Zuo X., He S., Li D., Peng C., Huang Q., Song S., Fan C. (2010). Graphene Oxide-Facilitated Electron Transfer of Metalloproteins at Electrode Surfaces. Langmuir.

[17] Kröner A., Hirsch T. (2019). Current Trends in the Optical Characterization of Two-Dimensional Carbon Nanomaterials. Front. Chem..

[18] Gugasyan R., Velazquez C., Vidavsky I., Deck B.M., van der Drift K., Gross M.L., Unanue E.R. (2000). Independent Selection by I-Ak Molecules of Two Epitopes Found in Tandem in an Extended Polypeptide Antigen1. J. Immunol..

[19] Xie B., Sharp J.S. (2016). Relative Quantification of Sites of Peptide and Protein Modification Using Size Exclusion Chromatography Coupled with Electron Transfer Dissociation. J. Am. Soc. Mass Spectrom..

[20] Liu Q., Wang J., Boyd B.J. (2015). Peptide-Based Biosensors. Talanta.

[21] Olesen L.E., Ford M.G.J., Schmid E.M., Vallis Y., Babu M.M., Li P.H., Mills I.G., McMahon H.T., Praefcke G.J.K. (2008). Solitary and Repetitive Binding Motifs for the AP2 Complex α-Appendage in Amphiphysin and Other Accessory Proteins. J. Biol. Chem..

[22] Choulier L., Enander K. (2010). Environmentally Sensitive Fluorescent Sensors Based on Synthetic Peptides. Sensors.

[23] Munteanu I.G., Grădinaru V.R., Apetrei C. (2022). Sensitive Detection of Rosmarinic Acid Using Peptide-Modified Graphene Oxide Screen-Printed Carbon Electrode. Nanomaterials.

[24] Huang Y., Hu F., Zhao R., Zhang G., Yang H., Zhang D. (2014). Tetraphenylethylene Conjugated with a Specific Peptide as a Fluorescence Turn-On Bioprobe for the Highly Specific Detection and Tracing of Tumor Markers in Live Cancer Cells. Chem.–Eur. J..

[25] Wang C., Wang J., Liu D., Wang Z. (2010). Gold Nanoparticle-Based Colorimetric Sensor for Studying the Interactions of β-Amyloid Peptide with Metallic Ions. Talanta.

[26] Clarke S., Pinaud F., Beutel O., You C., Piehler J., Dahan M. (2010). Covalent Monofunctionalization of Peptide-Coated Quantum Dots for Single-Molecule Assays. Nano Lett..

[27] Feng T., Feng D., Shi W., Li X., Ma H. (2012). A Graphene Oxide-Peptide Fluorescence Sensor for Proteolytically Active Prostate-Specific Antigen. Mol. Biosyst..

[28] Yan X., Yang L., Wang Q. (2011). Lanthanide-Coded Protease-Specific Peptide–Nanoparticle Probes for a Label-Free Multiplex Protease Assay Using Element Mass Spectrometry: A Proof-of-Concept Study. Angew. Chem. Int. Ed..

[29] Zhao N., He Y., Mao X., Sun Y., Zhang X., Li C., Lin Y., Liu G. (2010). Electrochemical Assay of Active Prostate-Specific Antigen (PSA) Using Ferrocene-Functionalized Peptide Probes. Electrochem. Commun..

[30] Vanova V., Mitrevska K., Milosavljevic V., Hynek D., Richtera L., Adam V. (2021). Peptide-Based Electrochemical Biosensors Utilized for Protein Detection. Biosens. Bioelectron..

[31] Anter J., Tasset I., Demyda-Peyrás S., Ranchal I., Moreno-Millán M., Romero-Jimenez M., Muntané J., Luque de Castro M.D., Muñoz-Serrano A., Alonso-Moraga Á. (2014). Evaluation of Potential Antigenotoxic, Cytotoxic and Proapoptotic Effects of the Olive Oil by-Product “Alperujo”, Hydroxytyrosol, Tyrosol and Verbascoside. Mutat. Res. Toxicol. Environ. Mutagen..

[32] Cardinali A., Linsalata V., Lattanzio V., Ferruzzi M.G. (2011). Verbascosides from Olive Mill Waste Water: Assessment of Their Bioaccessibility and Intestinal Uptake Using an In Vitro Digestion/Caco-2 Model System. J. Food Sci..

[33] Casaburi I., Puoci F., Chimento A., Sirianni R., Ruggiero C., Avena P., Pezzi V. (2013). Potential of Olive Oil Phenols as Chemopreventive and Therapeutic Agents against Cancer: A Review of in Vitro Studies. Mol. Nutr. Food Res..

[34] Ciriminna R., Meneguzzo F., Fidalgo A., Ilharco L.M., Pagliaro M. (2016). Extraction, Benefits and Valorization of Olive Polyphenols. Eur. J. Lipid Sci. Technol..

[35] Cicerale S., Lucas L., Keast R. (2010). Biological Activities of Phenolic Compounds Present in Virgin Olive Oil. Int. J. Mol. Sci..

[36] Del Carlo M., Amine A., Haddam M., della Pelle F., Fusella G.C., Compagnone D. (2012). Selective Voltammetric Analysis of O-Diphenols from Olive Oil Using Na2MoO4 as Electrochemical Mediator. Electroanalysis.

[37] Yang C., Denno M.E., Pyakurel P., Venton B.J. (2015). Recent Trends in Carbon Nanomaterial-Based Electrochemical Sensors for Biomolecules: A Review. Anal. Chim. Acta.

[38] Alessandri S., Ieri F., Romani A. (2014). Minor Polar Compounds in Extra Virgin Olive Oil: Correlation between HPLC-DAD-MS and the Folin-Ciocalteu Spectrophotometric Method. J. Agric. Food Chem..

[39] Paradiso V.M., Clemente A., Summo C., Pasqualone A., Caponio F. (2016). Towards Green Analysis of Virgin Olive Oil Phenolic Compounds: Extraction by a Natural Deep Eutectic Solvent and Direct Spectrophotometric Detection. Food Chem..

[40] Ricciutelli M., Marconi S., Boarelli M.C., Caprioli G., Sagratini G., Ballini R., Fiorini D. (2017). Olive Oil Polyphenols: A Quantitative Method by High-Performance Liquid-Chromatography-Diode-Array Detection for Their Determination and the Assessment of the Related Health Claim. J. Chromatogr. A.

[41] Ferro M.D., Santos S.A.O., Silvestre A.J.D., Duarte M.F. (2019). Chromatographic Separation of Phenolic Compounds from Extra Virgin Olive Oil: Development and Validation of a New Method Based on a Biphenyl HPLC Column. Int. J. Mol. Sci..

[42] Apetrei C., Rodríguez-Méndez M.L., de Saja J.A. (2005). Modified Carbon Paste Electrodes for Discrimination of Vegetable Oils. Sens. Actuators B Chem..

[43] Bounegru A.V., Apetrei C. (2021). Evaluation of Olive Oil Quality with Electrochemical Sensors and Biosensors: A Review. Int. J. Mol. Sci..

[44] Combination of an E-Nose, an e-Tongue and an e-Eye for the Characterisation of Olive Oils with Different Degree of Bitterness-PubMed. https://pubmed.ncbi.nlm.nih.gov/20172102/.

[45] Munteanu I.G., Apetrei C. (2021). A Review on Electrochemical Sensors and Biosensors Used in Chlorogenic Acid Electroanalysis. Int. J. Mol. Sci..

[46] Sudesh, Kumar N., Das S., Bernhard C., Varma G.D. (2013). Effect of Graphene Oxide Doping on Superconducting Properties of Bulk MgB _2_. Supercond. Sci. Technol..

[47] He Q., Sudibya H.G., Yin Z., Wu S., Li H., Boey F., Huang W., Chen P., Zhang H. (2010). Centimeter-Long and Large-Scale Micropatterns of Reduced Graphene Oxide Films: Fabrication and Sensing Applications. ACS Nano.

[48] Ma M., Fan X.P., Dai Z., Liu X., Xu S.C., Wei J., Shi S., Chen G.P. (2012). Graphene Oxide Modified DNA Electrochemical Biosensors. Appl. Mech. Mater..

[49] Liu J., Fu S., Yuan B., Li Y., Deng Z. (2010). Toward a Universal “Adhesive Nanosheet” for the Assembly of Multiple Nanoparticles Based on a Protein-Induced Reduction/Decoration of Graphene Oxide. J. Am. Chem. Soc..

[50] Çiplak Z., Yildiz N., Çalimli A. (2015). Investigation of Graphene/Ag Nanocomposites Synthesis Parameters for Two Different Synthesis Methods. Fuller. Nanotub. Carbon Nanostructures.

[51] Ghosh T.K., Gope S., Mondal D., Bhowmik B., Mollick M.R., Maity D., Roy I., Sarkar G., Sadhukhan S., Rana D. (2014). Assessment of Morphology and Property of Graphene Oxide-Hydroxypropylmethylcellulose Nanocomposite Films. Int. J. Biol. Macromol..

[52] Adochitei A., Drochioiu G. (2011). Rapid Characterization of Peptide Secondary Structure by FT-IR Spectroscopy. Rev. Roum. Chim..

[53] Arul A., Sivagnanam S., Dey A., Mukherjee O., Ghosh S., Das P. (2020). The Design and Development of Short Peptide-Based Novel Smart Materials to Prevent Fouling by the Formation of Non-Toxic and Biocompatible Coatings. RSC Adv..

[54] López De La Paz M., Goldie K., Zurdo J., Lacroix E., Dobson C.M., Hoenger A., Serrano L. (2002). De Novo Designed Peptide-Based Amyloid Fibrils. Proc. Natl. Acad. Sci. USA.

[55] Eckhart K.E., Holt B.D., Laurencin M.G., Sydlik S.A. (2019). Covalent Conjugation of Bioactive Peptides to Graphene Oxide for Biomedical Applications. Biomater. Sci..

[56] Rothschild K.J. (2016). The Early Development and Application of FTIR Difference Spectroscopy to Membrane Proteins: A Personal Perspective. Biomed. Spectrosc. Imaging.

[57] Surewicz W.K., Mantsch H.H., Chapman D. (1993). Determination of Protein Secondary Structure by Fourier Transform Infrared Spectroscopy: A Critical Assessment. Biochemistry.

[58] Adhikari B., Nanda J., Banerjee A. (2011). Pyrene-Containing Peptide-Based Fluorescent Organogels: Inclusion of Graphene into the Organogel. Chem. Weinh. Bergstr. Ger..

[59] Chronopoulou L., Di Nitto A., Papi M., Parolini O., Falconi M., Teti G., Muttini A., Lattanzi W., Palmieri V., Ciasca G. (2021). Biosynthesis and Physico-Chemical Characterization of High Performing Peptide Hydrogels@graphene Oxide Composites. Colloids Surf. B Biointerfaces.

[60] Pwavodi P.C., Ozyurt V.H., Asir S., Ozsoz M. (2021). Electrochemical Sensor for Determination of Various Phenolic Compounds in Wine Samples Using Fe3O4 Nanoparticles Modified Carbon Paste Electrode. Micromachines.

[61] Li X., Gao Y., Xiong H., Yang Z. (2021). The Electrochemical Redox Mechanism and Antioxidant Activity of Polyphenolic Compounds Based on Inlaid Multi-Walled Carbon Nanotubes-Modified Graphite Electrode. Open Chem..

[62] Dinu A., Apetrei C. (2020). Voltammetric Determination of Phenylalanine Using Chemically Modified Screen-Printed Based Sensors. Chemosensors.

[63] Apetrei I.M., Apetrei C. (2016). Voltammetric Determination of Melatonin Using a Graphene-Based Sensor in Pharmaceutical Products. Int. J. Nanomedicine.

[64] Bounegru A.V., Apetrei C. (2021). Development of a Novel Electrochemical Biosensor Based on Carbon Nanofibers–Cobalt Phthalocyanine–Laccase for the Detection of p-Coumaric Acid in Phytoproducts. Int. J. Mol. Sci..

[65] Dăscălescu D., Apetrei C. (2022). Development of a Novel Electrochemical Biosensor Based on Organized Mesoporous Carbon and Laccase for the Detection of Serotonin in Food Supplements. Chemosensors.

[66] Abrha T., Pal R., Saini R.C. (2017). A Study on Voltametric Electro-Kinetic Mechanism of Catechol at l-Glutamic Acid-Carbon Paste Sensor. J. Surf. Sci. Technol..

[67] Gil E., Enache T., Oliveira-Brett A. (2013). Redox Behaviour of Verbascoside and Rosmarinic Acid. Comb. Chem. High Throughput Screen..

[68] Enache T., Oliveira-Brett A. (2013). Phenol and Para-Substituted Phenols Electrochemical Oxidation Pathways. J. Electroanal. Chem..

[69] Zhuang W.-R., Wang Y., Cui P.-F., Xing L., Lee J., Kim D., Jiang H.-L., Oh Y.-K. (2019). Applications of π-π Stacking Interactions in the Design of Drug-Delivery Systems. J. Control. Release.

[70] Vilian A.T.E., Chen S.-M. (2015). Preparation of Carbon Nanotubes Decorated with Manganese Dioxide Nanoparticles for Electrochemical Determination of Ferulic Acid. Microchim. Acta.

[71] Apetrei I., Apetrei C. (2019). Development of a Novel Biosensor Based on Tyrosinase/Platinum Nanoparticles/Chitosan/Graphene Nanostructured Layer with Applicability in Bioanalysis. Materials.

[72] Apetrei I.M., Apetrei C. (2016). Amperometric Biosensor Based on Diamine Oxidase/Platinum Nanoparticles/Graphene/Chitosan Modified Screen-Printed Carbon Electrode for Histamine Detection. Sensors.

[73] Dinu A., Apetrei C. (2022). Quantification of Tyrosine in Pharmaceuticals with the New Biosensor Based on Laccase-Modified Polypyrrole Polymeric Thin Film. Polymers.

[74] Apetrei I.M., Apetrei C. (2018). A Modified Nanostructured Graphene-Gold Nanoparticle Carbon Screen-Printed Electrode for the Sensitive Voltammetric Detection of Rutin. Measurement.

[75] Pérez-Gregorio R., Soares S., Mateus N., de Freitas V. (2020). Bioactive Peptides and Dietary Polyphenols: Two Sides of the Same Coin. Molecules.

[76] Buitimea-Cantúa N.E., Gutiérrez-Uribe J.A., Serna-Saldívar S.O. (2018). Phenolic-Protein Interactions: Effects on Food Properties and Health Benefits. J. Med. Food.

[77] Caporale A., Adorinni S., Lamba D., Saviano M. (2021). Peptide–Protein Interactions: From Drug Design to Supramolecular Biomaterials. Molecules.

[78] Gruschwitz F.V., Klein T., Catrouillet S., Brendel J.C. (2020). Supramolecular Polymer Bottlebrushes. Chem. Commun..

[79] Yang J., An H.-W., Wang H. (2021). Self-Assembled Peptide Drug Delivery Systems. ACS Appl. Bio Mater..

[80] Thalassinos K., Pandurangan A.P., Xu M., Alber F., Topf M. (2013). Conformational States of Macromolecular Assemblies Explored by Integrative Structure Calculation. Structure.

[81] Zhu P., Zhao Y. (2019). Cyclic Voltammetry Measurements of Electroactive Surface Area of Porous Nickel: Peak Current and Peak Charge Methods and Diffusion Layer Effect. Mater. Chem. Phys..

[82] Bounegru A.V., Apetrei C. (2022). Simultaneous Determination of Caffeic Acid and Ferulic Acid Using a Carbon Nanofiber-Based Screen-Printed Sensor. Sensors.

[83] Gunache (Roșca) R.O., Bounegru A.V., Apetrei C. (2021). Determination of Atorvastatin with Voltammetric Sensors Based on Nanomaterials. Inventions.

[84] Tran M.T., Tran H.V. (2022). Verbascoside Extracted from Clerodendrum Inerme: A Natural Monomer for the Fabrication of a Sensitive Electrochemical Cu(II) Sensor. J. Chem. Res..

[85] Matos P., Paranhos A., Batista M.T., Figueirinha A. (2022). Synergistic Effect of DIBOA and Verbascoside from Acanthus Mollis Leaf on Tyrosinase Inhibition. Int. J. Mol. Sci..

[86] Ribeiro G.D.S., Carneiro A.D.A., Martins D.H.N., Simeoni L.A., Silveira D., Magalhães P.O., Fonseca-Bazzo Y.M. (2020). Determination of Harpagoside in Harpagophytum Procumbens DC Tablet’s Using Analytical Method by High Performance Liquid Chromatography. Eclética Quím. J..

[87] Araujo P. (2009). Key Aspects of Analytical Method Validation and Linearity Evaluation. J. Chromatogr. B.

[88] Gustavo González A., Ángeles Herrador M. (2007). A Practical Guide to Analytical Method Validation, Including Measurement Uncertainty and Accuracy Profiles. TrAC Trends Anal. Chem..

[89] Gali L., Bedjou F. (2019). Antioxidant and Anticholinesterase Effects of the Ethanol Extract, Ethanol Extract Fractions and Total Alkaloids from the Cultivated Ruta Chalepensis. S. Afr. J. Bot..

[90] Akar Z., Küçük M., Doğan H. (2017). A New Colorimetric DPPH• Scavenging Activity Method with No Need for a Spectrophotometer Applied on Synthetic and Natural Antioxidants and Medicinal Herbs. J. Enzyme Inhib. Med. Chem..

[91] Zielińska D., Turemko M. (2020). Electroactive Phenolic Contributors and Antioxidant Capacity of Flesh and Peel of 11 Apple Cultivars Measured by Cyclic Voltammetry and HPLC–DAD–MS/MS. Antioxidants.

[92] Munteanu I.G., Apetrei C. (2022). Tyrosinase-Based Biosensor—A New Tool for Chlorogenic Acid Detection in Nutraceutical Formulations. Materials.

[93] Munteanu I.G., Apetrei C. (2022). Assessment of the Antioxidant Activity of Catechin in Nutraceuticals: Comparison between a Newly Developed Electrochemical Method and Spectrophotometric Methods. Int. J. Mol. Sci..

[94] Bounegru A.V., Apetrei C. (2022). Sensitive Detection of Hydroxytyrosol in Extra Virgin Olive Oils with a Novel Biosensor Based on Single-Walled Carbon Nanotubes and Tyrosinase. Int. J. Mol. Sci..

[95] Arteaga J.F., Ruiz-Montoya M., Palma A., Alonso-Garrido G., Pintado S., Rodríguez-Mellado J.M. (2012). Comparison of the Simple Cyclic Voltammetry (CV) and DPPH Assays for the Determination of Antioxidant Capacity of Active Principles. Mol. Basel Switz..

[96] Rodriguez-Mendez M.L., Apetrei C., Gay M., Medina-Plaza C., de Saja J.A., Vidal S., Aagaard O., Ugliano M., Wirth J., Cheynier V. (2014). Evaluation of Oxygen Exposure Levels and Polyphenolic Content of Red Wines Using an Electronic Panel Formed by an Electronic Nose and an Electronic Tongue. Food Chem..

[97] Alipieva K., Korkina L., Orhan I.E., Georgiev M.I. (2014). Verbascoside—A Review of Its Occurrence, (Bio)Synthesis and Pharmacological Significance. Biotechnol. Adv..

[98] Vertuani S., Beghelli E., Scalambra E., Malisardi G., Copetti S., Toso R.D., Baldisserotto A., Manfredini S. (2011). Activity and Stability Studies of Verbascoside, a Novel Antioxidant, in Dermo-Cosmetic and Pharmaceutical Topical Formulations. Molecules.

